# KLVFF Functionalized Graphene Oxide for Aβ_42_ Peptide Electrical Detection: A Promising Nanomaterial for the Development of Alzheimer's Disease Diagnostic Devices

**DOI:** 10.1002/smll.202503488

**Published:** 2025-06-16

**Authors:** Viviana Scuderi, Rita Turnaturi, Simona Filice, Simona Crispi, Giuseppe Di Natale, Giuseppina Sabatino, Damiano Ricciarelli, Giuseppe Fisicaro, Giuseppe Pappalardo, Antonino La Magna, Silvia Scalese

**Affiliations:** ^1^ Consiglio Nazionale delle Ricerche ‐ Istituto per la Microelettronica e Microsistemi (CNR‐IMM) Ottava Strada n.5 Catania 95121 Italy; ^2^ Consiglio Nazionale delle Ricerche ‐ Istituto di Cristallografia (CNR‐IC) Via Paolo Gaifami n.18 Catania 95126 Italy

**Keywords:** alzheimer, biosensing, graphene oxide, KLVFF, β‐amyloid

## Abstract

Alzheimer's disease (AD) is a neurodegenerative disorder with a high negative impact in terms of cost and social issues. New kinds of noninvasive, reliable, easy‐to‐use, and cheap biosensors are highly desired. In this work, the use of graphene oxide (GO) sheets functionalized with the peg_4_‐KLVFF peptide conjugate is reported as a sensing layer for the detection of Aβ_42_ protein, a key AD biomarker. An optimized synthetic protocol provides a suitable GO/peptide‐functionalized layer without extensively modifying the planar structure of GO. The nature of the bonds involved in the functionalization process is highlighted through Fourier Transform Infrared Spectroscopy (FTIR) and X‐ray photoelectron spectroscopy (XPS) analyses. The covalently functionalized material (GO@peg_4_‐KLVFF) is deposited by dielectrophoresis (DEP) between two metal electrodes, forming the sensitive layer. The devices show a selective and linear electrical response as a function of Aβ_42_ concentrations. The selectivity toward Aβ_42_ is validated using two different scrambled sequences of the amyloid‐β peptides (Aβ_42s_, Aβ_40s_) as well as the Tau 26–44 peptide. The different electrical behaviors are discussed in detail and explained by simulating, at the molecular level, the interaction of the different Aβ peptides with GO and KLVFF. Potential chemical interactions are explored, including charge transfer between the peptides and GO.

## Graphical abstract

An electrical transduction sensor (GO@peg_4_‐KLVFF) deposited by DEP, based on graphene oxide, suitably functionalized for the detection of Aβ_42_, one of the main Alzheimer's disease biomarkers is proposed. The detection activity is established on the measurement of the electrical resistance change induced by the specific interaction occurring between the sensing layer and biomarker.

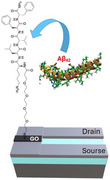

## 1 Introduction

Alzheimer's disease (AD) is a neurodegenerative disorder with a significant impact on society. It is expected to increase in the coming years due to the aging of the population.^[^ ^1^ ^]^ At the molecular level, Alzheimer's is characterized by the development of extracellular aggregates of amyloid β‐peptide (Aβ) deposited around neurons (senile plaques), and by the formation of intraneuronal neurofibrillary tangles of the protein tau.^[^ ^2^ ^]^ Aβ plaques and tau tangles spread progressively into the brain, compromising its performance. So Aβ and tau proteins play a fundamental role in the early diagnosis of AD, as they are related to the initial progress of AD.^[^ ^3^ ^]^

Early diagnosis is an important factor in controlling the disease, and delaying its symptoms. Currently, AD diagnosis mainly relies on medical imaging techniques, such as magnetic resonance imaging and positron emission tomography, cognitive tests, and cerebrospinal fluid analysis. These tests are generally performed when symptoms are advanced, and brain structures are already compromised.^[^ ^4^ ^]^

In this contest, biosensors can bring many improvements to AD diagnosis. In fact, they are less expensive than neuroimaging techniques and psychological analysis, and they are conceived as noninvasive because they should be used on easily accessible biological samples including blood serum, urine, or saliva. They could provide early diagnoses, as their operation is based on the detection of biomarkers that can influence the early development of the disease. They can help in monitoring the disease's course on a larger number of patients, acquiring more data for the study of the AD early stages, an important aspect for the development (and monitoring) of different therapies.^[^ ^5^ ^]^

Two fundamental parts characterize biosensors: the detection layer, composed of a biological element (such as antibodies, antigens, DNA, RNA, or enzymes) that interacts specifically with the analyte, and the transducer, which converts the interaction into measurable signals (electrical, optical, electrochemical, etc…).^[^ ^6^ ^]^

New strategies based on biomarker detection have been explored in the last few years for AD diagnostics.^[^ ^5^, ^7^ ^]^ As an example the Aβ_42_/Aβ_40_ ratio has proven to have a strong concordance with AD diagnosis and progression monitoring.^[^ ^8^ ^]^ Not only the level of Aβ_42_, but also phosphorylated tau (p‐tau), and total tau (t‐tau) proteins start to change almost 10–15 years before the appearance of AD symptoms.^[^ ^9^ ^]^

Similarly to proteins, but distinguished by their smaller size, peptides are short chains of amino acid monomers with fewer than fifty amino acids. They are particularly appealing in the context of AD since it has been shown that a short peptide fragment of Aβ, namely the KLVFF pentapeptide, upon binding to Aβ can inhibit its aggregation.^[^ ^10^ ^]^ As an antifibrillogenic agent, the KLVFF peptide can act in several ways to interfere with Aβ fibril formation. It selectively binds to regions within full‐length Aβ, particularly the homologue core sequence Aβ (16‐20). Subsequent studies have reported that this ability can be also modulated by the experimental environment as well as peptide structure (position of amide and amino groups in the chain, halogenated peptides), and the present data seem promising for the development of peptide‐based drugs for the treatment of AD.^[^ ^11^ ^]^

The good affinity of KLVFF for Aβ monomers and oligomers underscores its importance in the early stages of amyloid aggregation and its potential as a target for therapeutic interventions aimed at preventing or disrupting amyloid plaque formation.^[^ ^12^ ^]^ This selective interaction offers a potential mechanism for modulating Aβ aggregation in AD. Recent efforts in molecular diagnostics have focused on developing devices for the sensitive detection of Aβ. An interesting strategy involves the development of biosensors, including optical, electrochemical, and surface plasmon resonance (SPR) sensors, and peptide‐based ligands that specifically recognize Aβ species. The detection effectiveness of most graphene oxide‐based Aβ biosensors considers specific antibodies as recognition elements of the biological biomarker.^[^ ^13^ ^]^ An antibody‐based biosensor is counteracted by an antibody's high cost of production, molecular instability, and struggling to distinguish between target‐specific binding versus nonspecific interactions that might arise from a not univocal immobilization of the antibody over the sensing platform. Most of these issues can be easily overcome by using peptides as Aβ recognition elements: their relatively low cost of production, high stability, and easier yet selective chemical methods to functionalize the transducer material, make the peptide‐functionalized graphene oxide (GO) layers a suitable choice for the construction of efficient Aβ_42_’s detection devices.

In this work, GO sheets were functionalized with KLVFF peptide and were used as a sensing layer for the detection of Aβ_42_, highlighting the potentiality of the system. The choice of using GO and KLVFF depends on the properties of the two materials. GO offers a large surface area and, due to the presence of various oxygen‐containing functional groups, provides numerous reactive sites useful for peptide immobilization. The KLVFF peptide, on the other hand, was shown to selectively interact with Aβ. Importantly, a peg_4_ spacer (peg_4_ = 15‐amino‐7,7,10,13‐tetraoxapentadecanoic acid) was introduced at the N‐terminus of the KLVFF sequence to distance the peptide‐recognizing moiety from the GO surface.

The ratio between GO and peg_4_‐KLVFF was optimized to get satisfactory functionalities for the biomarker detection, without significantly modifying the planar structure of GO. The nature of the bonds involved in the functionalization process was inferred through Fourier Transform Infrared Spectroscopy (FTIR) and X‐ray photoelectron spectroscopy (XPS) analysis. The final functionalized material (GO@peg_4_‐KLVFF) was used to develop a resistive device, where the active layer was deposited by dielectrophoresis (DEP) between two metallic electrodes. The devices showed a selective and linear response with Aβ_42_ concentration in the investigated range. The selectivity toward Aβ_42_ was validated by comparing the results obtained for two different scrambled Aβ peptides (Aβ_42s_, Aβ_40s_), i.e., molecules that contain the same amino acids but ordered in a different sequence with respect to the native peptides. In addition, to further stress the selectivity issues, the GO@peg_4_‐KLVFF response toward the AD‐related Tau 26–44 peptide fragment was also recorded. The different electrical behaviors were discussed in detail and satisfactorily explained by simulating, at the molecular level, the interaction of the different Aβ peptides with GO and KLVFF. In this framework, some potential chemical interactions were explored, including charge transfer between the protein and GO.

## 2 Results and Discussion

### 2.1 Chemical Characterization

In order to find the best ratio between added functionalities and GO, while preserving the morphological features of the GO sheets, different GO/peg_4_‐KLVFF concentration ratios were tested: a) GO 0.5 mg mL^−1^ + peg_4_‐KLVFF 1 µg mL^−1^; b) GO 0.5 mg mL^−1^ + peg_4_‐KLVFF 10 µg mL^−1^; c) GO 0.5 mg mL^−1^ + peg_4_‐KLVFF 100 µg mL^−1^; d) GO 0.5 mg mL^−1^ + peg_4_‐KLVFF 1000 µg mL^−1^. The GO@peg_4_‐KLVFF dispersions were dropped on Si substrates, placed in a desiccator for 24 h, and then observed by SEM. The functionalized nanomaterials appeared well dispersed in the case of samples (a), (b) and (c), while some very large agglomerates were observed in sample (d) (see Figure S1, Supporting Information). In the last case, the concentration of peptide is probably excessive, inducing aggregation phenomena and not allowing a complete dispersion of the functionalized GO in water.^[^ ^14^ ^]^

**Figure 1:**
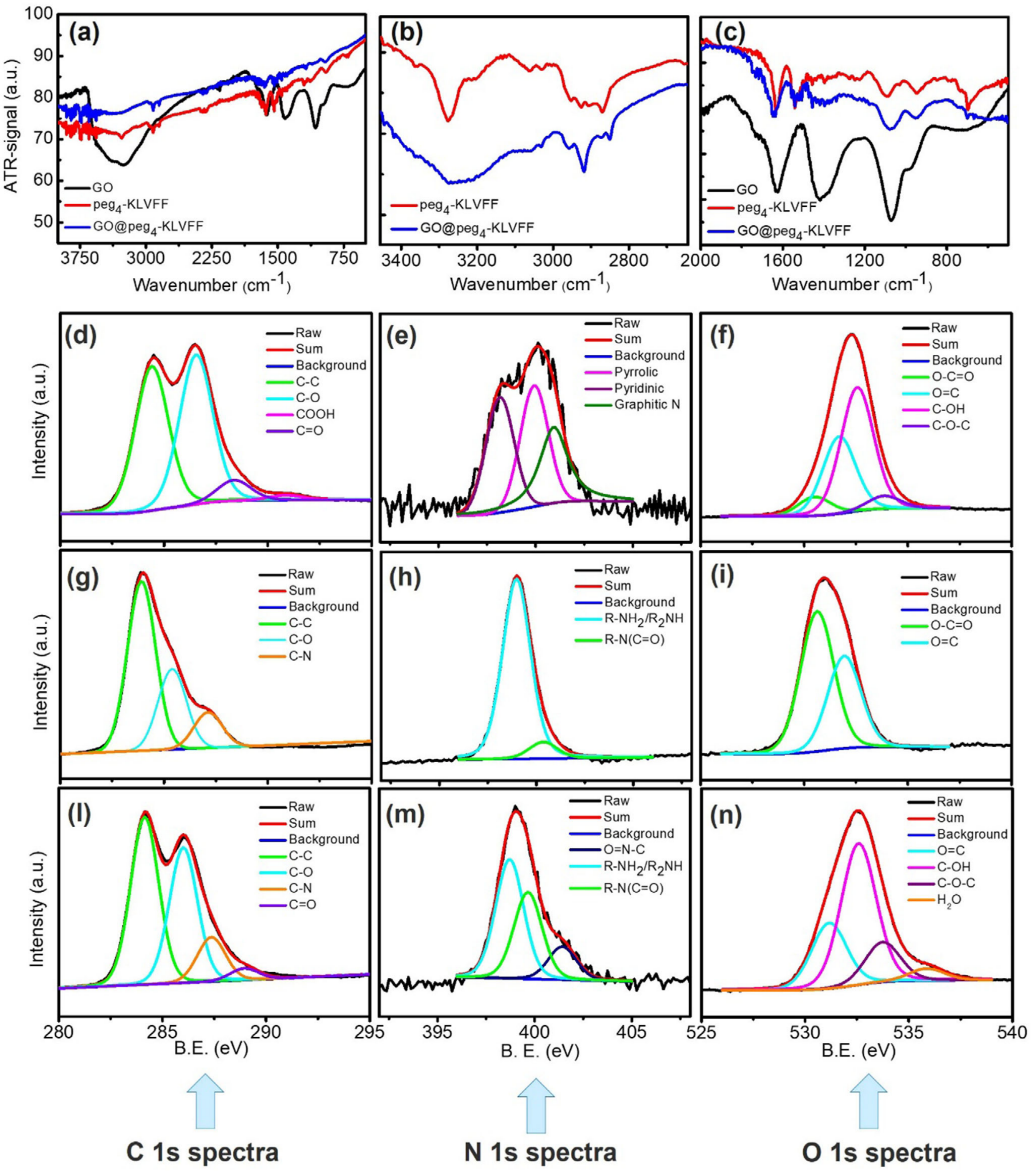
a) ATR FT‐IR spectra of GO (black line), peg_4_‐KLVFF peptide (red line), and GO@peg_4_‐KLVFF peptide (blue line). Same spectra in the b) 3500–2500 cm^−1^ and c) 2000–500 cm^−1^ wavenumber ranges. For a better visualization of the peaks, the spectra were vertically shifted arbitrarily to avoid overlapping. In Figure 1b, the GO spectrum was not reported to highlight the difference between the other two spectra. XPS spectra of: C1s for d) GO, g) peg_4_‐KLVFF, l) GO@peg_4_‐KLVFF; N1s for e) GO, h) peg_4_‐KLVFF, m) GO@peg_4_‐KLVFF; O1s for f) GO, i) peg_4_‐KLVFF, n) GO@peg_4_‐KLVFF. Black continuous lines indicate the acquired spectra, red lines are the fits of the spectra, blue lines are the subtracted baseline, and the other colored lines identify the different contributions obtained by deconvolution of the peaks. The GO@peg_4_‐KLVFF ratio was 0.5 mg mL^−1^/100 µg mL^−1^).

Attenuated Total Reflection (ATR)FTIR analysis was performed to verify the successful functionalization of GO with the peptide and to identify the nature of the bindings involved. **Figure** **1**a reports the ATR FT‐IR spectra of GO (black line), peg_4_‐KLVFF peptide (red line), and GO functionalized with the peg_4_‐KLVFF peptide (blue line) in the 4000–500 cm^−1^ wavelength range.

A wide band corresponding to stretching vibrations of the hydroxyl group (OH group, from adsorbed water) occurs in the wavenumber range 3700–3000 cm^−1^ for the GO sample. This band is partially visible in the GO@peg_4_‐KLVFF sample (Figure 1a). To better observe the variation of the peaks in the different spectra, these were divided into two intervals 3500–2500 cm^−1^ and 2000–500 cm^−1^, and reported in Figure 1b,c. From the analysis, we neglect the spectral region between 2500–2000 cm^−1^, because it is influenced by typical features related to environmental carbon dioxide.

Both peg_4_‐KLVFF and GO@peg_4_‐KLVFF show peaks between 3000 and 2800 cm^−1^ associated with the symmetric and asymmetric CH‐stretching vibrations. As reported in **Table** **1**, these peaks vary in both shape and position.

**Table 1:** Principal ATR FT‐IR peaks were detected for GO, peg_4_‐KLVFF, and GO@peg_4_‐KLVFF.

Material	Wavelength [cm^−1^]	Chemical bond vibration mode
GO	3700–3300	O─H stretching (water adsorbed)
	1710	C─O stretching of C═O in COOH
	1626	C═C aromatic
	1415	C─O carboxy
	1069	C─O epoxy
	976	C=C stretching
peg_4_‐KLVFF	3276	N─H stretching
	2953–2869	C─H stretching symmetric and asymmetric
	1632.23	CO stretching
	1537.47	C─N, stretching
	1069	C─O epoxy
	974	C═C stretching
	696	N─H out of plane bending
GO@peg_4_‐KLVFF	2959–2850	C─H stretching symmetric and asymmetric
	1642.23	CO stretching
	1530.47	C─N, stretching
	1069	C─O epoxy
	974	C═C stretching

The shifts of the peaks to lower and higher values suggest a change in the electron distribution of the molecular bonds, probably due to strong interaction with GO.

Although attenuated in intensity, GO characteristic peaks (Figure 1c, Table 1) are still maintained in the GO@peg_4_‐KLVFF sample, except for the peak at 1710 cm^−1^ related to the C═O in ─COOH group, as well as the peak at 1415 cm^−1^ due to the carboxyl C─O decreases significantly. Indeed, both peg_4_‐KLVFF and GO@peg_4_‐KLVFF show the characteristic amide bands at 1632 and 1537 cm^−1^ and 1642 and 1530 cm^−1^, respectively. In Figure 1b, the peg_4_‐KLVFF shows a sharp peak at 3276 cm^−1^ and at 696 cm^−1^ associated with N‐H group vibrations not detectable in the GO@peg_4_‐KLVFF sample.

GO@peg_4_‐KLVFF sample can be considered as the sum of the contributions from the GO‐only and peptide‐only samples. Based on the ATR FT‐IR data, it therefore appears that the covalent bond between GO and the peptide has occurred, and it involves the carboxyl group of GO and the amine group of the peptide.

To characterize the chemical composition of the GO sheets, with or without peptide functionalization, XPS analysis was performed, allowing us to identify the chemical nature of the bonds involved in the GO functionalization process. Figure 1 shows the C1s, N1s, and O1s XPS spectra of pristine GO (d,e,f), peg_4_‐KLVFF (g,h,i) and GO@peg_4_‐KLVFF (l,m,n).

In particular, the black continuous lines indicate the acquired spectra, red lines are the fits of the spectra, blue lines are the subtracted baselines, and the other colored lines are the deconvolution of the peaks. In Figure 1d for the GO sample, C1s peak was deconvolved using four contributions: C─C bonds at 284.5 eV (green line), C─O at 286.5 eV (cyan line), C═O at 288.4 eV (violet line), and COOH at 290.4 eV (magenta line). For the peg_4_‐KLVFF sample (Figure 1g) the peak related to the COOH group is not present, whereas another peak at lower energy (287.1 eV), related to C─N bonds (orange line), is found. For the functionalized sample (GO@peg_4_KLVFF), Figure 1l the peak related to the COOH group is not present, while peaks related to the C─C, C─O, C═O, C─N bonds can be observed.

Figure 1e,h,m shows the N1s peak obtained for pristine GO, peg_4_‐KLVFF, and GO@peg_4_‐KLVFF samples, respectively. For GO the N1s peak intensity is small, indicating that it is related to contamination. It was deconvoluted using three contributions: pyridinic at 399.2 eV (purple line), pyrrolic at 400.9 eV (magenta line), and graphitic N at 401.9 eV (olive line).^[^ ^15^ ^]^ For peg_4_‐KLVFF the N1s peak shows the R‐NH_2_ or R_2_NH signal at 399.0 eV (cyan line) and R‐N(C═O) at 400.4 eV (green line).^[^ ^16^ ^]^ For the GO@peg_4_‐KLVFF sample, the N1s show the R‐NH_2_ or R_2_NH peak at 399.7 eV (cyan line), R‐N(C═O) at 400.7 eV (green line) and O═N─C at 402.4 eV (navy line).

In Figure 1f for the GO sample, O1s XPS spectrum displays four contributions: O─C═O at 530.5 eV (green line), O═C at 531.7 eV (cyan line), C─OH at 532.6 eV (magenta line) and C─O─C at 533.8 eV (violet line). The peg_4_‐KLVFF sample (Figure 1i) shows only two components: O─C═O at 530.6 eV (green line) and O═C at 531.9 eV (cyan line). In the case of the GO@peg_4_‐KLVFF sample (Figure 1n), the deconvolution of O1s peak shows the suppression of the O─C═O peak, the presence of O═C at 531.2 eV (cyan line), C─OH at 532.6 eV (magenta line), C─O─C at 533.7 eV (violet line) peaks and an additional peak at 535.9 eV (orange line). Typically peaks in the O1s band at binding energies between 535 and 536 eV are associated with surface interaction effects with water.^[^ ^17^ ^]^ Small variations in the position of the deconvolutions are attributable to the different nature and complexity of the two structures.

**Tables** **2**,**3** and **4** report the relative weight (%) of each component of the C1s, O1s, and N1s XPS peaks, respectively, as achieved by peak deconvolution processes. For the functionalized sample (GO@peg_4_‐KLVFF), it is interesting to observe the absence of the COOH group (Table 2) and the reduction of the amino component (R‐NH_2_/R_2_NH) from 91.5% to 49.9% in favor of the amide one (R‐N(C═O)) from 8.5% to 36.3% (Table 4), which are the groups involved in the formation of the bond between GO and peg_4_‐KLVFF.

**Table 2:** Relative amounts (%) of different bond types resulting from the C1s peak deconvolution.

Bond type	GO	peg_4_‐KLVFF	GO@peg_4_‐KLVFF
% C─C	45.2	59.3	46.7
% C─O	46.8	28.2	37.7
% C─N	–	12.5	12.3
% C═O	6.5	–	3.3
% COOH	1.5	–	–

**Table 3:** Relative amounts (%) of different bond types resulting from the O1s peak deconvolution.

Bond type	GO	peg_4_‐KLVFF	GO@peg_4_‐KLVFF
% O─C═O	8.2	60.2	–
% O═C	33.2	39.8	23.4
% C─OH	53.2	–	44.9
% C─O─C	5.5	–	28.4
% H_2_O	–	–	3.3

**Table 4:** Relative amounts (%) of different bond types resulting from the N1s peak deconvolution.

Bond type	GO	peg_4_‐KLVFF	GO@peg_4_‐KLVFF
% pyridinic	33.8	–	–
% pyrrolic	35.5	–	–
% graphitic N	30.7	–	–
% R‐NH_2_/R_2_NH	–	91.5	49.9
% R‐N(C═O)	–	8.5	36.3
% O═N─C	–	–	13.8

XPS data confirm what is already observed by ATR FT‐IR analyses, i.e., the functionalization occurred between the carboxyl group of GO and the amino group of the peptide.

### 2.2 Dielectrophoretic Deposition of the Sensing Layer, Sensitivity and Selectivity Tests

**Figure 2:**
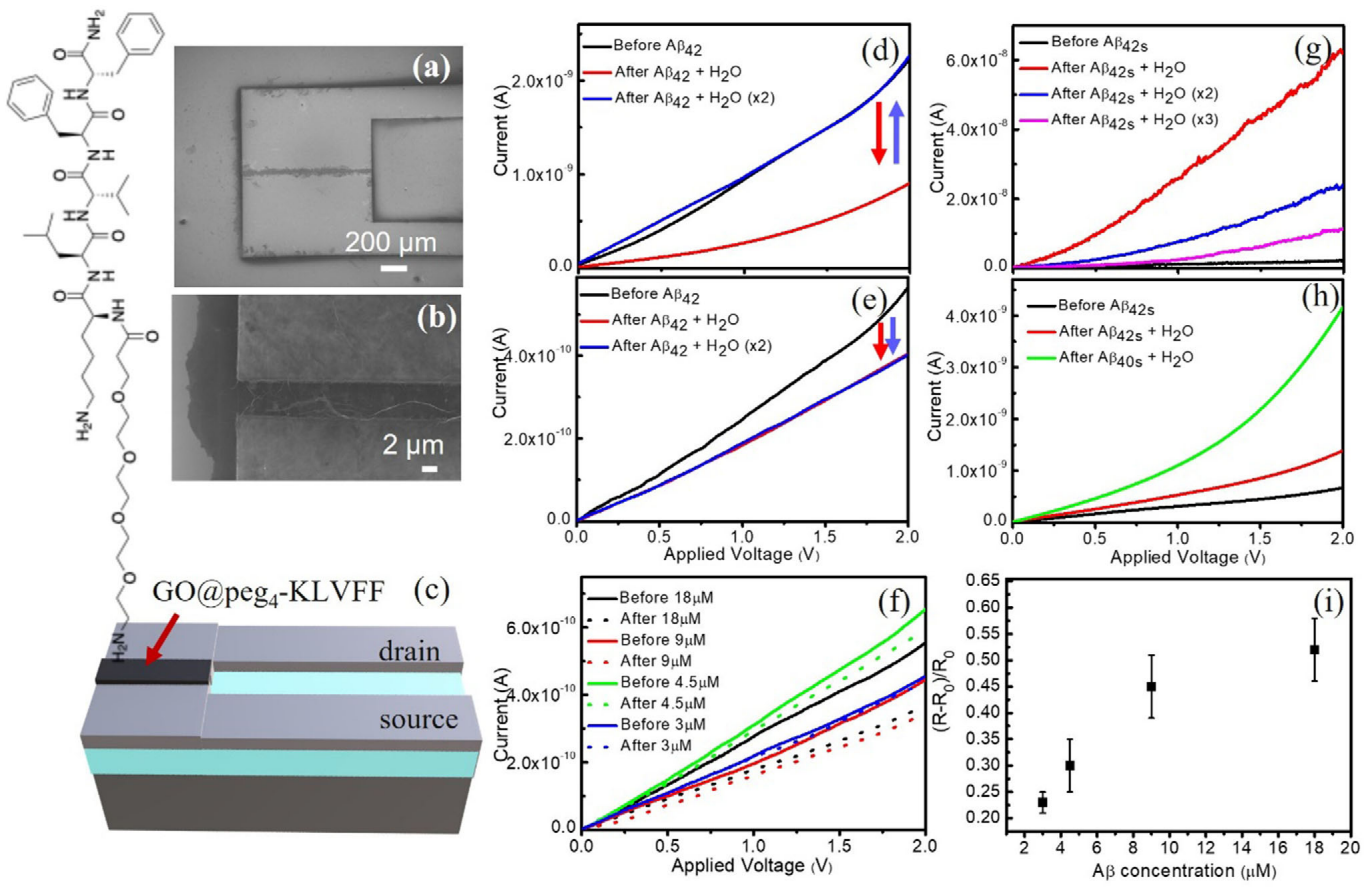
SEM images of the electrode region after the deposition of a GO@peg_4_‐KLVFF layer by DEP: a) overview, b) a magnification of the region between the electrodes, c) a schematic of the functionalized device. *I–V* curves obtained for d) GO and e) GO@peg_4_‐KLVFF layers before (black line) and after the interaction with Aβ_42_ followed by single wash (red line) or double wash (blue line). f) *I–V* curves for GO@peg_4_‐KLVFF samples after the interaction with Aβ_42_ at different concentrations. For each device/concentration, the *I–V* curve before (solid line) and after (dotted line) the interaction with Aβ_42_ is shown. i) Sensitivity values (ΔR/R_0_) versus Aβ_42_ concentration. *I–V* curves for g) pristine GO sample before (black line) and after the interaction with Aβ_42s_ followed by one (red line), two (blue line) or three (magenta line) washing processes; h) GO@peg_4_‐KLVFF sample before (black line) and after the interaction with Aβ_42s_ (red line) and Aβ_40s_ (green line) followed by abundant washing in deionized water. A SEM image at a higher magnification of the GO@peg_4_‐KLVFF deposited by DEP is reported in Figure S2 (Supporting Information).

To test the sensing properties of the functionalized material, several samples were deposited by dielectrophoresis (DEP), as described in the Experimental section, using GO (0.5 mg mL^−1^) and GO@peg_4_‐KLVFF dispersions obtained according to the synthesis protocols employing different GO/peptide concentration ratios. Preliminary tests were conducted using both the GO@peg_4_‐KLVFF (ratio: 0.5 mg mL^−1^/10 µg mL^−1^) and GO@peg_4_‐KLVFF (ratio: 0.5 mg mL^−1^/100 µg mL^−1^) samples. The best results in terms of electrical response to the analyte were obtained using the GO@peg_4_‐KLVFF (ratio: 0.5 mg mL^−1^/100 µg mL^−1^) sample, therefore from now on, we discuss the data obtained from the measurements carried out using this GO@peg_4_‐KLVFF derivative. **Figure** **2** shows SEM images of the region between the electrodes after the deposition of a GO@peg_4_‐KLVFF layer, at lower (a) and higher (b) magnification, respectively. A schematic of the functionalized device is shown in Figure 2c

A continuous and homogeneous film was deposited along the entire length of the electrodes, showing some folds, typical of the morphology of GO layers, with no evidence of large aggregates. Contact angle measurements were performed on GO and GO@peg_4_‐KLVFF samples, after being deposited by DEP between the electrodes. For GO and GO@peg_4_‐KLVFF samples, a contact angle of about 61° and 76° was measured, respectively (see Figure S3, Supporting Information). Therefore, the functionalization seems responsible for a higher hydrophobicity of the system.

After electrical characterization, the unfunctionalized GO and the GO@peg_4_‐KLVFF samples were exposed to Aβ_42_, as reported in Figure 2d,e, respectively. 1 µL of Aβ_42_ solution with a concentration of 9 µm was dropped in the region between the electrodes, left to interact for 5 min, and then washed in deionized water. As a general protocol: after sample deposition on the electrode and rinsing, the material was subjected to a first reading of the current and washed again with deionized water (twice) before to a second reading. Aβ_42_ was diluted in a mixture of water and hexafluoroisopropanol (HFIP) (ratio 4:1) to reduce aggregation phenomena, and the measured pH value was 5.5. In fact, when the pH approaches the isoelectric point (pI, pH 5.1), the absence of charges removes the repulsion forces and favors the formation of intermolecular contacts involved in the aggregation process.

The two devices show a different initial current (black lines), despite having maintained the same GO concentration in the two samples. This different behavior could depend on the deposition process. In fact, since the size and the shape of GO and GO@peg_4_‐KLVFF systems are different, they could respond differently to the same dielectrophoretic field and, therefore, a different total amount of material could be deposited between the electrodes. Since the peg_4_‐KLVFF interacts chemically with the GO, another possible effect could be the exchange of charges between the GO and the peptide, leading to the modification of the electrical response.^[^ ^18^ ^]^

In the case of pristine GO (Figure 2d) the electrical signal of the device (black lines) decreases after exposure to Aβ_42_ followed by washing in deionized water (red line). After further rinsing, the electrical response of the device (blue line) returns to the initial value observed before exposure to Aβ_42_ and does not change anymore after further washing. In the case of GO@peg_4_‐KLVFF (Figure 2e) the electrical current of the device (black lines) decreases after exposure to Aβ_42_ followed by washing in deionized water (red line) and it is not modified by further rinsing in deionized water (blue line). This different behavior suggests that in the case of an unfunctionalized GO layer, Aβ_42_ is only weakly adsorbed over the GO surface (probably through London forces, Van der Waals interactions, and hydrogen bonds) and that abundant rinsing in water is sufficient to remove Aβ_42_ from the GO surface. In the case of the GO@peg_4_‐KLVFF layer, instead, Aβ_42_ interacts mainly with peg_4_‐KLVFF through the KLVFF sequential part that they share (see Figure S4, Supporting Information). This interaction is stronger and more stable than the previous one and abundant rinsing in water is not sufficient to move Aβ_42_ away from the functionalized GO.

As reported above, the KLVFF sequence corresponds to residues 16–20 of the Aβ full‐length peptide. A wealth of literature data supports a selective interaction occurring through the binding of the KLVFF motif to complementary regions within the Aβ peptides. This underscores the importance of this peptide sequence in setting up molecular systems aimed at detecting amyloid peptides for theragnostic purposes.^[^ ^12^, ^19^ ^]^

Once it was verified that Aβ_42_ interacts with the functionalized system through peg_4_‐KLVFF and that the interaction is stable, new functionalized devices with similar initial electrical properties were exposed to Aβ_42_ with different concentrations in the range between 3–18 µm, and results were reported in Figure 2f. For each device/concentration, the *I–V* curves after the interaction with the Aβ_42_ (dotted lines) show a reduction in the slope compared to the values before the interaction (solid lines). The reduction is proportional to the concentration of Aβ_42_ that the GO@peg_4_‐KLVFF was exposed to. The sensitivity is expressed in terms of ΔR/R_0_, where R_0_ is the channel resistance before the interaction with Aβ_42_ and ΔR = R‐R_0_, where R is the channel resistance after the interaction with Aβ_42_. The values of R and R_0_ were acquired at 2 V, and the results are reported in Figure 2i. All samples were reproduced in triplicate and each point in the graph was calculated by averaging the values obtained from the *I–V* curves measured for three samples at the same concentration. The exposure to Aβ_42_ induces an increase in the channel resistance as the concentration of the Aβ_42_ is increased. The increase is linear in the range between 3–9 µm, while at higher values up to 18 µm ΔR/R_0_ reaches a plateau. The response can be addressed to the electrical charge transfer phenomena induced on the GO@peg_4_‐KLVFF by the Aβ_42_ molecules. For higher Aβ_42_ values (e.g., 18 µm), the active sites of the sensing layer are saturated and, therefore, a plateau is reached.

Similarly, concentrations lower than 1 µm, up to 500 nm, were investigated. At these Aβ_42_ values, the signal variation is comparable to electrical noise. Also in this case, an increase in the active area and, therefore, of active sites could improve the capability of the device, as well as the sensitivity of the active layer. In the literature authors reported resistive devices with a sensitive layer of 4.5 mm^2^, which means an active layer more than 1000 times greater with respect to the one reported in our work.^[^ ^20^ ^]^ The use of a larger sensitive region is expected to improve the sensitivity of our sensing layer, especially if we consider the presence of a nanostructured material (GO) that increases more than linearly the effective surface area exposed to the biomarkers. Another interesting factor is represented by the interaction time between the active layer and the species to be detected. For Aβ_42_ detection by reduced GO, an interaction time of 30 or 40 min is reported.^[^ ^20^, ^21^ ^]^ In our case recognition between peg_4_‐KLVFF and Aβ_42_ is very fast, and a few minutes are sufficient to saturate the interaction between the two species.

Further experiments were carried out to investigate the specificity of the commercial GO and GO@peg_4_‐KLVFF devices toward the Aβ_42_ detection. Both systems were exposed to a scrambled sequence of Aβ_42_, i.e., Aβ_42s_, as reported in Figure 2g,h, while the GO@peg_4_‐KLVFF devices were exposed to scrambled Aβ_40s_, as shown in Figure 2h. 1 µL of a solution of Aβ_42s_ or Aβ_40s_ with a concentration 9 µm was deposited in the region between the electrodes and left to interact for 5 min and washed in deionized water. As explained before (Figure 2d,e), the two devices show a different initial current (black lines), because of normal fluctuations due to the deposition process performed on two materials that are not identical; however, this difference is not a great issue since the sensitivity values obtained for each device are normalized to its own initial resistance. In the case of GO alone (Figure 2f) the electrical signal of the device (black lines) increases after exposure to Aβ_42s_ followed by washing in abundant deionized water (red line). After further washing in deionized water, the electrical current of the device decreases (blue line) and it decreases even more after further washing (magenta line). If the GO@peg_4_‐KLVFF (Figure 2h) is considered, the response of the device to the interaction with Aβ_42s_ is similar to that recorded in the presence of pristine GO. The current increases after exposure to Aβ_42s_ and remains higher even after washing in deionized water (red line). The same behavior is observed in the presence of Aβ_40s_ (green line), with an increase of the current in the device after interaction with it, followed by washing in deionized water. In both cases the increase in the electrical signal is attributable to the presence of Aβs residues on the surface, not completely removed by the washing processes. The same output (the increase of the current) for the two samples suggests that in both cases the interaction takes place between the scrambled Aβ peptides and the GO. It should be pointed out that the scrambled sequences of the Aβ peptides were different (see Figure S4, Supporting Information) thus suggesting that the electrical response we observed is specific for the Aβ_42_. Indeed, if we compare the response of the GO sample after the interaction with Aβ_42_ reported in Figure 2d and the GO sample after the interaction with Aβ_42s_ reported in Figure 2g, they show an opposite trend. In the first case, the current decreases after the interaction with Aβ_42_, in the second case the current increases after the interaction with Aβ_42s_. The pI (isoelectric point) value, the pH at which Aβ_42_ has a neutral charge, is approximately 5.1 which indicates that Aβ_42_ is slightly negatively charged at pH 5.5. However, it is known that the amino acid position and pK are responsible for the net charge at each pH.^[^ ^22^ ^]^ So, even if the Aβ_42_ and Aβ_42s_ solutions have the same pH, the different amino acid positions along the chain might influence the net charge, as well as the aggregation process and the interaction with GO.

We wanted to further test the response of the GO@peg_4_‐KLVFF layer by exposing it to another AD‐related compound. An eventual negative response would be indicative of the lack of cross‐reactivity with markers other than Aβ. A recent comprehensive mass spectrometry screening, carried out on cerebrospinal fluid (CSF) samples from AD patients, revealed increased levels of Tau peptide fragments, namely the Tau 25–44 and Tau 6–23 belonging to the N‐terminal domain of the full‐length parent protein, with respect to healthy controls.^[^ ^23^ ^]^ So, we carried out additional experiments considering the Tau 26–44 peptide as another kind of AD biomarker. We performed the measurements under identical experimental conditions as for the Aβ_42_ biomarker. Interestingly, the response of the devices is similar to that already observed in the presence of scrambled Aβs (see Figure S5, Supporting Information). In particular, for pristine GO the current values of the device (black lines) increase after exposure to Tau 26–44 followed by washing in abundant deionized water (red line). After further washing in deionized water, the electrical current decreases (blue line). In the case of GO@peg4‐KLVFF, the response of the device to the interaction with Tau 26–44 is similar to that recorded in the presence of pristine GO, confirming that there is no specific interaction between Tau and the functional peptide.

### 2.3 Effect of Aβ_42_ Aggregation on the Electrical Response

Increasing evidence has suggested that the formation and propagation of misfolded aggregates of Aβ_42_ causes AD.^[^ ^24^ ^]^ Thus, the device's ability to detect Aβ_42_ of different sizes was tested. GO@peg_4_‐KLVFF devices were exposed to a freshly prepared Aβ_42_ solution (Aβ_42_), a 40‐days aged Aβ_42_ solution (Aβ_42_(40 days)), and 4 months aged Aβ_42_ solution (Aβ_42_(4 months)) stored at 4 °C, to induce the aggregation of Aβ_42_. All solutions had an initial concentration of 9 µm. Interaction times and rinses are equal to those described in previous tests.

**Figure 3:**
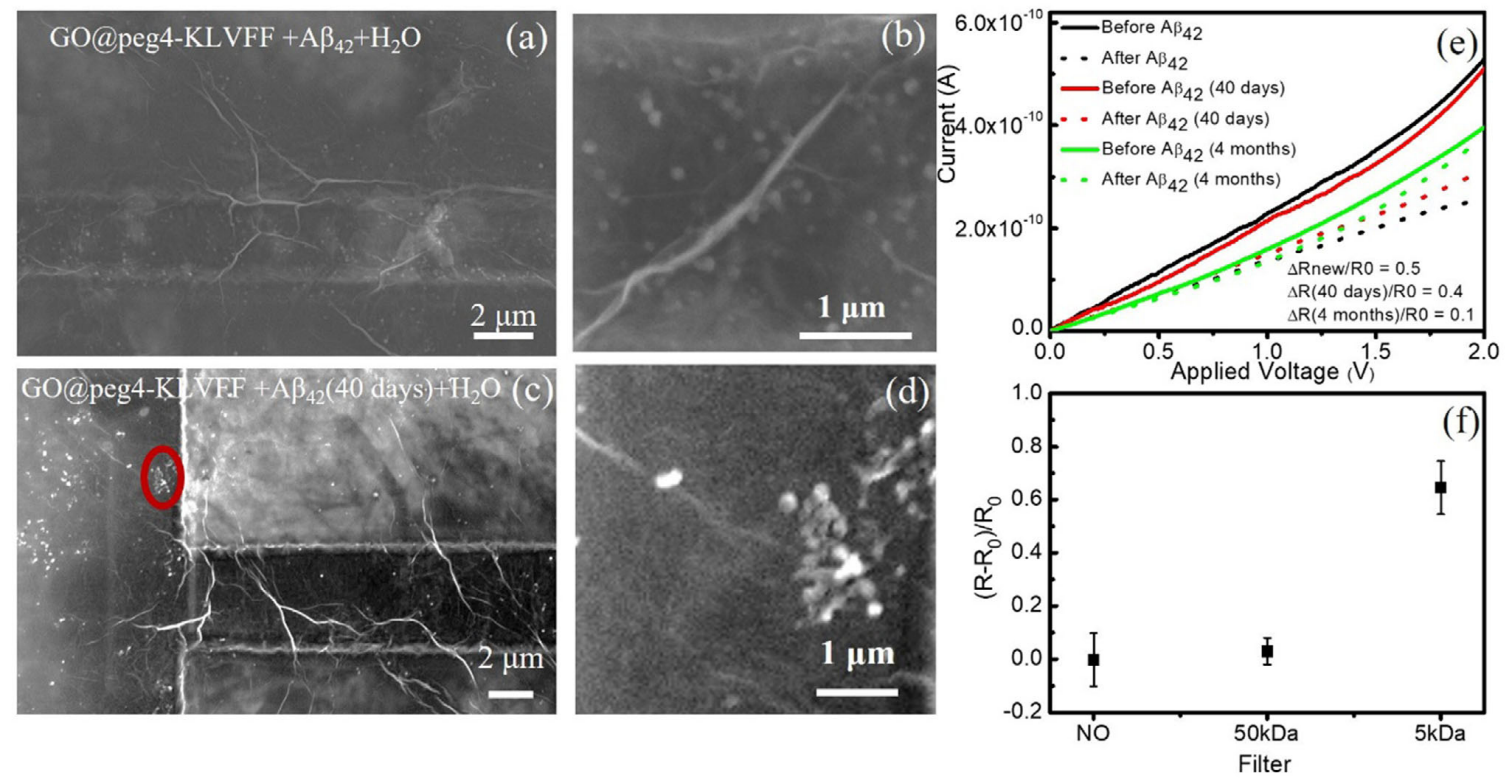
SEM images of the devices GO@peg_4_‐KLVFF after the interaction with: a) Aβ_42_ and c) Aβ_42_(40 days) solution. A higher magnification is shown in the panel b,d), respectively. e) *I–V* curves for GO@peg_4_‐KLVFF samples before (solid lines) and after (dotted lines) the interaction with Aβ_42_ (black line), Aβ_42_(40 days, red line), and Aβ_42_ (4 months, green line), at the same concentration value (9 µm). f) Sensitivity values (ΔR/R_0_) versus Aβ_42_ concentration.

**Figure** **3**a,c shows the SEM images of the GO@peg_4_‐KLVFF devices after the exposition to Aβ_42_ and Aβ_42_(40 days), respectively, after washing in H_2_O.

In the region between the electrodes, SEM analyses show mainly the presence of agglomerates (clear circular structures) with dimensions between 60–120 nm in the device exposed to the Aβ_42_ solution (a higher magnification is shown in Figure 3b), while in the device exposed to the Aβ_42_(40 days) solution (Figure 3c), the agglomerates have a larger size in the range 100–500 nm, as highlighted in the red circle (a higher magnification is shown in Figure 3d). The size of the folded Aβ_42_ monomer is ≈3–4 nm, and the oligomer or protofibril will be above 5 nm.^[^ ^24^ ^]^ Therefore, in both cases at pH 5.5 we observed protofibrils of different average sizes.

Figure 3e shows the *I–V* curves for the devices before (solid lines) and after (dotted lines) the interaction with Aβ_42_ (black line), Aβ_42_(40 days, red line), and Aβ_42_(4 months, green line) 9 µm. For all devices, the *I–V* curves after the interaction with the Aβ_42_ show a reduction in the slope of the curves compared to the values before the interaction. The reduction is greater in the presence of the Aβ_42_ (dotted black line), and lower in the other two cases. In particular, the sensitivity expressed in terms of ΔR/R_0_ is 0.5 (Aβ_42_new), 0.4 (Aβ_42_(40 days)), and 0.1 (Aβ_42_(4 months)), for the *I–V* curves reported in Figure 3e.

To get an idea of how the device might respond in the presence of Aβ in its aggregated forms, new measurements were performed using aged Aβ_42_ samples. We resorted to a 4‐months‐aged Aβ sample that was subjected to a centrifugation process (10 000 rpm, 3 min, 25 °C), in the presence of two different porous septa with cut‐offs of 5 kDa and 50 kDa. This process allows to separate monomers from oligomers and larger aggregates. In the presence of the 5 kDa porous septum, after the centrifugation process, the resulting solution is essentially enriched with monomers. The centrifuged and uncentrifuged solutions were deposited in triplicate on different devices. The sensitivity (mean value) expressed in terms of ΔR/R_0_ is reported in Figure 3f. We observe that for the uncentrifuged solution and for the centrifuged solution with a 50 kDa cut‐off septum, the sensitivity (taking into account the error) is less than 0.1. For the 5 kDa cut‐off solution, the sensitivity is approximately 0.6, suggesting that the KLVFF peptide moiety recognizes and interacts predominantly with monomeric Aβ_42_ species. Oligomers and larger aggregates seem to be quite stable when put in contact with the KLVFF‐functionalized sensing layer and, thus, do not contribute significantly to the electrical current changes. In fact, it is possible that in the presence of aggregated structures, the KLVFF sequence, that the Aβ shares with the peptide, is no longer exposed and, therefore, it is not available for recognition.

Under normal conditions, Aβ peptides are generated regularly by the enzymatic action of secretases on amyloid precursor protein (APP). This fact denotes a probable physiological action of Aβ that is still being studied intensively. Some data indicate that Aβ in its monomeric form has a neuroprotective action by supporting the neuron with an insulin‐like action promoting glucose uptake.^[^ ^25^ ^]^ Aβ’s turnover is strictly controlled, however, in AD, this balance is disrupted. Thus, Aβ monomers can aggregate and form toxic oligomers and amyloid plaques, which are hallmark features of AD. Therefore, detecting, monitoring, and understanding the dynamics of Aβ monomer concentrations are essential for evaluating disease progression and developing potential therapeutic strategies for AD.^[^ ^26^ ^]^

### 2.4 Adsorption of Protein Monomers and Peptides onto GO: Computational Analysis

**Figure 4:**
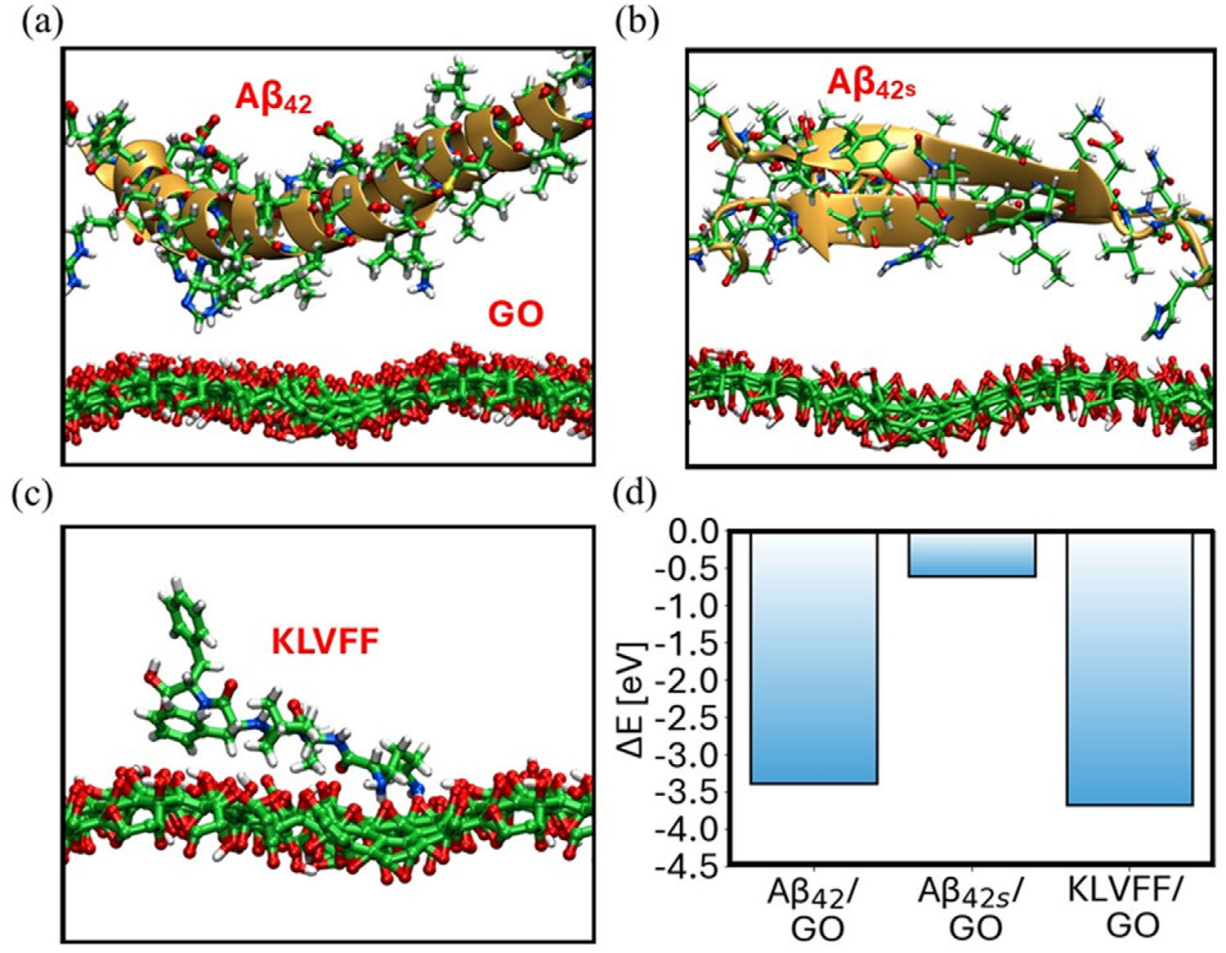
Physisorption of a) Aβ_42_, b) Aβ_42s_, and c) KLVFF onto GO, with their respective energy profiles in d).

To elucidate the varying trends observed in the *I–V* curves at the molecular level, we investigated the adsorption of protein monomers and peptides onto GO, considering the various physicochemical interactions. In our approach we initially predicted possible structures of protein monomers, starting from their peptide sequences and we then simulated the interaction of monomers with GO, performing structural relaxations with a state‐of‐the‐art machine‐learning interatomic potential (see the Methodological Section for additional details). Our analysis addresses both unfunctionalized GO and peg_4_‐KLVFF functionalized GO.^[^ ^27^ ^]^ The protein structural prediction delivers an Aβ_42_ monomer, i.e., **Figure** **4**a, characterized with a α‐helix configuration, in agreement with some experimental studies, and an Aβ_42s_ monomer with a β‐sheet domain, i.e., Figure 4b.^[^ ^28^ ^]^ We notice that the extension of the α and β domains may vary with solvents and temperature, with monomer regions sometimes even acquiring a random coil configuration, depending on the external conditions.^[^ ^29^ ^]^

Figure 4a–c illustrates the physisorption of protein monomers and of the KLVFF onto GO structures. The physisorption energies, presented in Figure 4d, were determined by calculating the energy difference between the physisorption systems, isolated proteins, and GO. These results reveal that Aβ_42_ binds stronger to GO if compared to Aβ_42s_ with respective energies of −3.4 and −0.6 eV for Aβ_42s_. Interestingly also KLVFF strongly binds with the material. An increased interaction of Aβ_42_ compared to Aβ_42s_ is further evidenced by a 10 ps molecular dynamics simulation of the physisorption systems. In the case of Aβ_42_, the α‐helix closely approaches GO. On the other hand, for Aβ_42s_, the protein also gets closer to GO but significantly less than the former with the interaction between the sequences highlighted by the gold arrows preserved throughout the entire dynamics, manifesting also a high tendency toward aggregation.

To obtain more quantitative results on the protein monomers aggregation, we predict the structure of dimers in Figure S6a,b (Supporting Information) and we compute the monomer aggregation energies, i.e., Figure S6d (Supporting Information), as energy differences between dimers and monomers. To broaden the energetics horizon, we also calculate the interaction between Aβ_42_ and KLVFF (Figure S6c, Supporting Information) occurring within identical amino acid sequences, also reported in Figure S6d (Supporting Information). The dimer structure of Aβ_42_, i.e., Figure S6a (Supporting Information), highlights the formation of fybrils domains, proper of this class of systems, while the dimer structure of Aβ_42s_ shown in Figure S6b (Supporting Information) is mostly characterized by random coils.

The energetics of monomer aggregation in Figure S6d (Supporting Information) suggest that Aβ_42s_ have a stronger leaning toward clustering than Aβ_42_, featuring a protein‐protein interaction energy of −3.9 eV versus −2.5. Notably, KLVFF exhibits a high affinity for GO, and, at the same time, the peptide interacts strongly with Aβ_42_. This confirms its role in anchoring Aβ_42_ to the GO substrate, preventing its aggregation.

**Figure 5:**
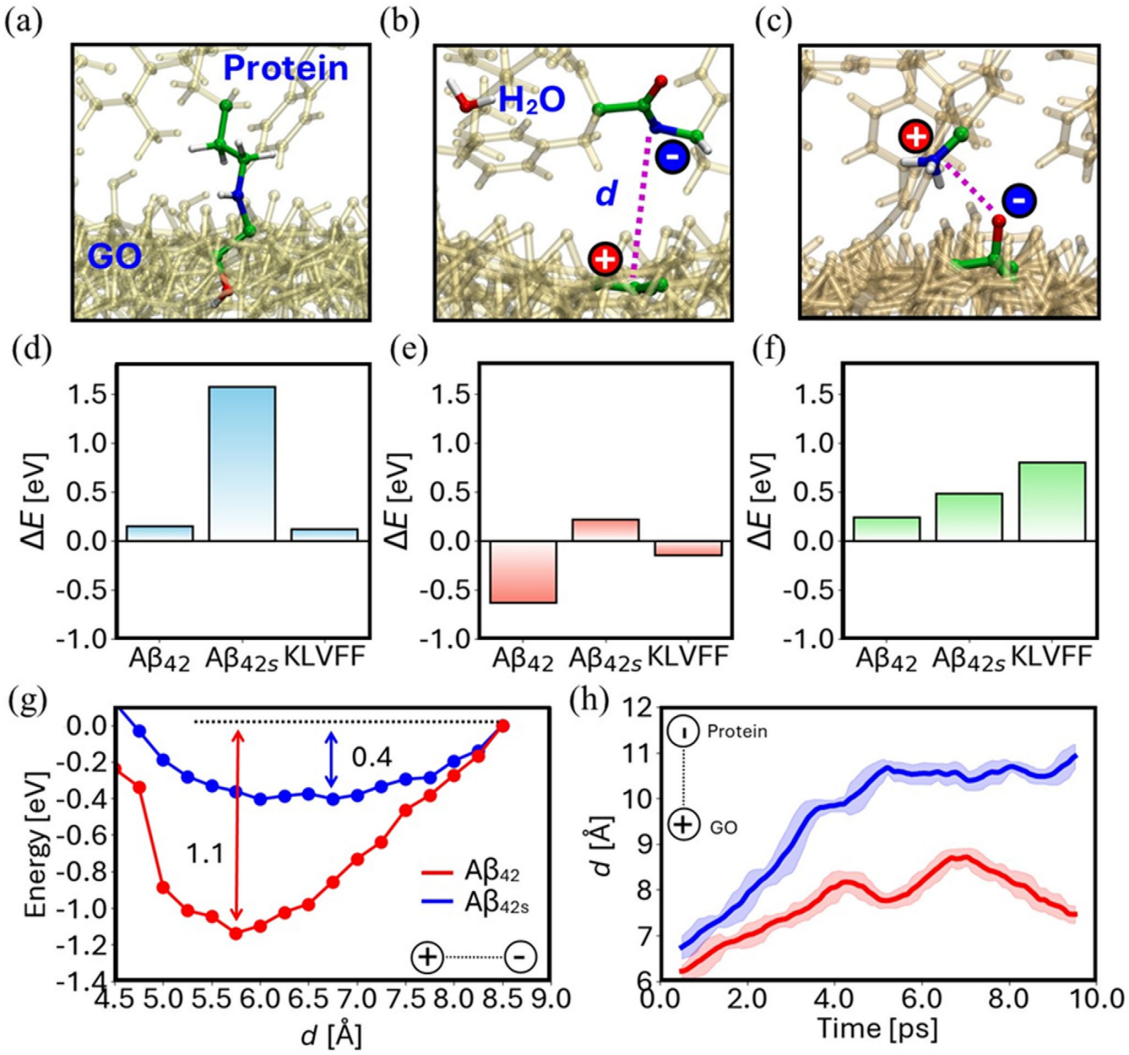
Chemisorption of Aβ_42_, Aβ_42s_, and KLVFF onto GO with corresponding chemisorption energies: a) GO epoxide (C─O─C) opening via lysine nucleophilic attack, with energies in (d); b) GO hydroxy (C─OH) proton extraction by an amidic group, leading to water evolution, with energies in e); c) GO hydroxy (C─OH) proton transfer to lysine, with energies in (f). g) Energy versus protein‐GO electron–hole charge separation (labelled as *d*) for the case in panel (b), from a linear transit calculation. h) Time evolution of protein‐GO electron–hole charge separation for the case in panel (b) over 10 ps of molecular dynamics at 300 K.

We further explored possible protein‐GO chemisorption interactions, reported in **Figure** **5**, which likely play a major role in affecting both the mobility and the concentration of charge carriers in GO and thus the measured *I–V* curves. From local chemistry analysis, epoxide (C─O─C) groups are more likely to undergo nucleophilic attack reactions due to their ring strain, as shown in Figure 5a. In contrast, hydroxyl (C─OH) groups are more prone to generate electrostatic interactions with the protein upon proton exchange with the former, as illustrated in Figure 5b,c. Specifically, C─OH proton extraction from the protein may leave GO with a positive charge and favor the release of a water molecule, i.e., Figure 5b, while C─OH proton release may negatively charge the material, i.e., Figure 5c.

Considering previous knowledge in the current modulation of graphene‐based devices through interfacing with proteins, we observe that different adsorption behaviors, depending on the protein, can significantly influence the device's electrical properties.^[^ ^30^ ^]^ Specifically, if protein‐GO interactions are strong, intrinsic charge mobility may be reduced, leading to a decrease in device current. Conversely, if adsorption facilitates the release of free charges into GO, charge carrier concentration increases, thereby enhancing the device current.

We observe that chemisorption energies, computed as energy differences between chemisorbed and physiosorbed structures, associated with a nucleophilic attack, proton extraction, and proton release, shown in Figure 5d–f, consistently favor Aβ_42_. This suggests, in alignment with the physisorption energies, a stronger overall tendency of Aβ_42_ to interact with the material across all examined interactions. Aβ_42s_, on the other hand, as shown in Figure 5e,f, is unlikely to engage in nucleophilic interactions, as indicated by the corresponding chemisorption energy of 1.7 eV, but it exhibits thermal accessible energies for charging the material, as reported in Figure 5e,f. KLVFF globally follows the same trend of Aβ_42_.

For the selected case in Figure 5b, we computed the protein‐GO electron–hole binding energies shown in Figure 5g. The results highlight that Aβ_42s_ enhances GO charge carriers through chemical interaction, as evidenced by its lower electron–hole binding energy ≈0.4 eV compared to Aβ_42_ ≈1.1 eV. This lower binding energy indicates weaker charge confinement at the GO‐protein interface, facilitating charge separation and increasing the number of free charge carriers in graphene oxide. In contrast, the higher binding energy of Aβ_42_ confines charges at the interface, contributing to restricting their mobility and limiting the generation of free charge carriers. This is further supported by the time evolution of protein‐GO charge separation distances in Figure 5h from molecular dynamics simulations. While Aβ_42_ maintains a stable charge separation of ≈7 Å, indicating strong electrostatic attraction, Aβ_42s_ shows a steady increase, exceeding 11 Å, reflecting weaker attraction. As a result, the residual charge on GO becomes free, acting as a charge carrier. With the general picture provided by simulations in mind, we can now draw some discussion on presented variations in device curves, experimentally detected for both the GO and GO@peg_4_‐KLVFF. Focusing on the unfunctionalized material, simulation results highlight that the interaction of Aβ_42_ with GO is stronger than the one of Aβ_42s_ for all simulated scenarios. Specifically, Aβ_42_ presents strong surface interactions that hamper the collection of extrinsic and intrinsic charge carriers, causing an overall decrease of the measured current (i.e., an increase in the material's resistivity), rationalizing the behavior in Figure 2d.

On the other hand, Aβ_42s_ has lower adsorption energies and an increased tendency for aggregation. However, its interaction with the substrate can generate charges at the interface with GO (from the results of Figure 5e,f). Eventually, weak binding interactions allow charge separation, freeing the residual charge on GO to act as a charge carrier. This behavior is likely to increase the measured current (i.e., decrease resistivity), as documented in Figure 2g. For the GO@peg_4_‐KLVFF, the impact on the *I–V* curves during interaction with Aβ_42_ and Aβ_42s_ remains unchanged and can be fully explained by the earlier discussion on unfunctionalized materials, albeit with some subtle differences. We notice that the KLVFF peptide presents a behavior that parallels with Aβ_42_, characterized by strong interactions with the material. This, likewise, contributes to a decrease in the amount of measured current compared to the unfunctionalized material, in line with experimental results in Figures 2d,e and 3e,f. On the other hand, its strong interaction at the same time with GO and Aβ_42_, documented in Figure 4, contributes to strongly anchoring the protein to the surface. As a matter of fact, when exposed to Aβ_42_, the current of GO@peg_4_‐KLVFF does not return to its initial state even after repeated washing, indicating a scenario where Aβ_42_ remained strongly attached to GO and its aggregation is totally prevented.

Finally, when aged solutions of Aβ_42_ are adsorbed onto GO@peg_4_‐KLVFF, the observed current decrease is smaller than that seen with fresh solutions, as shown in Figure 3a. This is likely due to Aβ_42_ forming aggregates in aged solution, as illustrated in Figure 4d–h, which, when exposed to GO, still possesses a high thermodynamic barrier for dissociation. For instance, the calculated barrier for the Aβ_42_ dimer is 2.5 eV. As a result, the probability of these oligomers chemisorbing onto the material within the time scale of the measurement is reduced.

## 3 Conclusion

In this work, the sensing properties of a GO@peg_4_‐KLVFF system toward one of the well‐known biomarkers of AD, Aβ_42_, have been highlighted. In particular, the GO/peg_4_‐KLVFF ratios have been optimized to obtain a well‐dispersed material that was deposited by DEP between two electrodes to obtain a homogeneous sensitive layer for electrical detection of Aβ_42_.

FTIR and XPS analysis confirmed that the chemical bond between GO and the peptide occurs through the carboxyl group of GO and the secondary amine of the peptide.

The produced resistive GO@peg_4_‐KLVFF layers were electrically characterized by performing *I–V* curves before and after Aβ_42_ and scrambled Aβ peptides exposure, and GO layers were used for comparison. The different electrical behaviors were discussed in detail and satisfactorily explained by simulating, at the molecular level, the interaction of the different Aβ species with GO and KLVFF. Theoretical results point out that Aβ_42_ generates stronger adsorption to GO than Aβ_42s_. The robust interfacial interactions between Aβ_42_ and GO likely impact charge carrier mobility, whereas Aβ_42s_, which interact in a milder way, contribute to an increase in the density of GO charge carriers.

A linear electrical response was observed as a function of the Aβ_42_ concentration in a range between 3–9 µm, with a slope changing for higher concentration values, reaching a plateau for Aβ_42_ concentrations of 18 µm, probably due to the saturation of the active sites of interaction. The sensitivity range is strictly related to the current size and geometry of the device and could be extended in the future by increasing the sensitive area of the device and the number of active sites of the functionalized layer.

At this stage, the GO@peg_4_‐KLVFF system has been demonstrated to be promising for the development of future AD diagnostics devices, due to the following remarkable properties: a great ability to detect Aβ_42_ monomers; the selectivity toward Aβ_42_ (the system responds specifically only in the presence of Aβ_42_, and not in the presence of scrambled structures containing the same amino acids arranged in a random order) or the other AD‐related Tau 26–44; an extremely fast response thanks to the interaction between the GO@peg_4_‐KLVFF system and the Aβ_42_, that occurs within a few minutes.

Finally, it is hopefully expected that the GO@peg_4_‐KLVFF construct would selectively respond to Aβ_40_ monomers. Such an event is particularly significant because it is known that in AD, changes in the plasma concentrations of the Aβ_40_/Aβ_42_ ratio occur long before clinical symptoms can be detected. The early determination of these changes at the molecular level is a key aspect for a timely diagnosis that would allow therapeutic interventions aimed at slowing down the rate of cognitive decline.

## 4 Experimental Section

### Materials

Commercially available reagents were used directly unless otherwise noted. Peptide‐grade N,N‐dimethylformamide (DMF), all Fmoc protected amino acids (Fmoc‐Lys‐OH, Fmoc‐Leu‐OH, Fmoc‐Val‐OH, Fmoc‐Phe‐OH), N,N′‐diisopropylcarbodiimide (DIC), Oxyma Pure, (benzotriazol‐1‐yloxy) tripyrrolidinophosphonium hexafluorophosphate (PyBOP), trifluoroacetic acid (TFA), triisopropylsilane (TIS), N,N‐diisopropylethylamine (DIPEA), diisopropyl ether (iPr_2_O), diethyl ether (Et_2_O), dichloromethane (DCM), 2‐propanol and HPLC‐grade acetonitrile (CH_3_CN), GO (O 50%) solution, 1‐ethyl‐3‐(3‐dimethylaminopropyl) carbodiimide hydrochloride (EDC), N‐hydroxy succinimide (NHS) were purchased from Sigma–Aldrich (Milan, Italy). Fmoc‐rink Amide AM resin and Fmoc‐NH‐(peg)₄─COOH were purchased from Iris Biotech (Germany).

Aβ_42_ native and Aβ_42_ scrambled sequence (Aβ_42s_) were purchased from Bachem (Switzerland), Aβ_40_ scrambled sequence (Aβ_40s_) was acquired from AnaSpec (USA). The synthesis of the Tau 26–44 peptide was reported elsewhere.^[^ ^31^ ^]^ The Aβ peptides were subjected to a disaggregation protocol before their use, (see Figure S2, Supporting Information).^[^ ^19b^ ^]^ Ultrapure water produced by Synergy UV equipment.

### Synthesis of peg_4_‐KLVFF

The peptide KLVFF was assembled starting from 0.30 mmol of Rink Amide AM resin (loading 0.35 mmol g^−1^, 100–200 Mesh) according to the Fmoc/tBu protocol by using the microwave‐assisted solid‐phase peptide synthesizer (MW‐SPPS) Liberty Blue 2.0 (CEM, Matthews, NC, U.S.A.), as previously reported.^[^ ^32^ ^]^

Fmoc‐(PEG)_4_‐OH (2 equiv.) was manually coupled offline, using PyBOP as a condensing agent in the presence of DIPEA (2 and 4 equiv. respectively). The cleavage of the peptide from the resin, with concomitant deprotection, was performed with TFA/TIS/H_2_O (95:2.5:2.5, 1 mL mixture/10 mg of resin) for 2.5 h, at room temperature under stirring. After filtration, the peptide was recovered by precipitation from the cleavage mixture with ice‐cold iPr_2_O.

The purity of the peptide was verified by reverse‐phase HPLC (RP‐HPLC) using a SHIMADZU LC‐20A chromatography system equipped with an SPD‐M20A photodiode array detector (detection at 222 and 254 nm) on a Kinetex XB‐C18 250 × 4.60 mm (100 Å pore size, 5 µm) column. The elution was achieved with a mobile phase consisting of water + 0.1% TFA (eluent A) and acetonitrile + 0.1% TFA (eluent B). The peptide was eluted at a flow rate of 1 mL min^−1^ according to the following protocol: isocratic 10% B in 3 min, gradient 10–100% B in 11 min (Rt = 10.1 min, (software LabSolution Ink)). The integrity of the peptide was assessed by LC/MS using a Thermo Fisher (Q‐Exactive). [obsd: m/z = 472.30 (M+2H)^2+^; Calcd. for C_48_H_78_N_8_O_11_ m/z = 472.30 (M+2H)^2+^. (Figure S7, Supporting Information).

### Synthesis of GO@peg_4_‐KLVFF

Peptide peg_4_‐KLVFF was covalently conjugated with GO by EDC/NHS protocol. EDC (0.02 mmol) and NHS (0.05 mmol) were added to a water dispersion of GO (0.5 mg mL^−1^) and sonicated for 60 min at room temperature. To the resulting mixture, aqueous peg_4_‐KLVFF (1000, 100, 10, 1 µg mL^−1^) was then added. After pH adjustment to 8, the mixture was stirred in the dark at room temperature overnight. The unbound peptide was removed from the GO dispersion by centrifugation at 13 000 rpm for 30 min. The obtained pellet was resuspended in water.

### Characterization of GO, peg_4_‐KLVFF and GO@peg_4_‐KLVFF

Fourier Transformation Infrared (FTIR) measurements were performed by a Jasco FT‐IR‐4700 spectrophotometer equipped with an Attenuated Total Reflection (ATR‐PRO ONE) with a diamond prism. Clamps ensure sample contact with the crystal. The solutions were deposited on commercial aluminum foil. X‐ray photoelectron spectroscopy (XPS) analysis was carried out using the PHI Genesis Multi‐Technique Scanning XPS system, with a monochromatic Al Kα (1486.6 eV) X‐ray beam and a 180° hemispherical electron energy analyzer. The system, equipped with a dual‐beam charge neutralization system, allows turnkey neutralization of all types of insulating samples.

Morphological characterization of the samples was performed using a field emission scanning electron microscope (Supra 35 FE‐SEM by Zeiss, Oberkochen, Germany).

The wettability of the surface was assessed by measuring the optical contact angle using a Dataphysics OCA 25 system (DataPhysics Instruments GmbH, Filderstadt, Germany) and a drop size of 1 µL.

### Dielectrophoresis

GO (0.5 mg mL^−1^) and GO@peg_4_KLVFF (0.5 mg mL^−1^ + 100 µg mL^−1^), dispersed in deionized water and diluted at 10 µL mL^−1^, were deposited via dielectrophoresis (DEP) between two Pt/Ti electrodes (1000 µm long strips, with a distance between electrodes of 4 µm) placed on SiO_2_/Si(100) substrate. DEP was the movement of polarizable particles exposed to a nonuniform electric field. The behavior of DEP force depends on the particle size, shape, and material, and, therefore, DEP parameters used for achieving a localized deposition of a specific kind of nanostructures must be chosen properly.

For this purpose an alternating electric field (Vpp = 20 V; ν = 100KHz) was applied between the electrodes kept immersed in the above‐described dispersions for a time of 20 min. All samples were reproduced in triplicate.^[^ ^33^ ^]^

### Electrical Measurements

Electrical characterization was performed by a Source Meter Unit (SMU), Keithley 6430, on dried samples, and the current values were acquired for a voltage range between −2 and 2 V. The work reports values between 0 and 2 V. Before and after exposure to the analyte to be detected (Aβ_42_, Aβ_42s_, Aβ_40s_, Tau 26–44), the samples deposited by DEP were electrically characterized by repeating the IV measurements four times in order to verify the electrical stability.

### Stability of the Sensing Film

The GO@peg_4_‐KLVFF solution was largely stable (about 4 months) if stored in a refrigerator at 4 °C. The stability of the functionalized material (GO@peg_4_‐KLVFF) deposited by DEP between the electrodes, with respect to storage conditions, was verified by monitoring the stability of the electrical response (resistance) as a function of time for two sets of samples: one stored in a refrigerator at 4 °C and one stored in a desiccator. The samples stored in a desiccator were stable at least for a week and after this time the resistance values start to increase for some of the samples. While for the samples stored in a refrigerator at 4 °C the measurements remain stable (within the experimental error) at least for 18 days. The resistance values for the two sets of samples as a function of time were shown in Tables S1 and S2 (Supporting Information).

### Computational Methodology

Interaction energies between Aβ_42_, Aβ_42s_, KLVFF, and GO were computed performing structural relaxations with the MACE‐MP machine learning interatomic potential, implemented in the Atomic Simulation Environment (ASE) python library, together with dispersion corrections.^[^ ^27a^ ^]^ The GO models were constructed by enlarging preexisting cell structures (with a 50% oxygen mass percentage) previously computed by some of us.^[^ ^34^ ^]^ In this study, GO nanosheets consisting of a total of 1070 atoms were constructed, characterized by 𝑎 and 𝑏 lattice parameters of 40.91 and 44.48 Å, respectively, and separated by an interlayer distance of 50 Å. The distribution of oxygen atoms between the epoxy and hydroxyl groups was designed to align with XPS measurements.^[^ ^35^ ^]^ The structures of Aβ_42_ and Aβ_42s_ monomers predicted by Alphafold were utilized starting from their respective amino‐acid sequence and providing the experimental structure of Aβ_42_ as a first guess.^[^ ^27^, ^28^ ^]^ In this context, an initial run was conducted using AlphaFold2 with the mmseqs2_uniref_env, unpaired_paired method and 48 recycles. Subsequently, refinement was carried out under the same computational parameters, utilizing the previous structure as a template. For the structural predictions of dimers, the same methodology was employed switching to AlphaFold2_multimer_v2, following the procedure reported in recent studies on Aβ_42_.^[^ ^36^ ^]^ The structural models with the highest pLDDT scores were collected and used to calculate the energies of monomer aggregation and interaction with GO, i.e., physisorption and chemisorption, with GO, performing geometry optimizations for the various systems. The linear transit calculations in Figure 5g were conducted using the same computational parameters as the structural relaxations, with the *d* distance from Figure 5b fixed at predetermined values. The molecular dynamics simulations in Figure 5h were initiated from the systems presented in Figure 5b, employing the same computational parameters as the structural relaxations. Additional details include a temperature of 300 K within an NVT ensemble and a timestep of 1 fs.

## Conflict of Interest

The authors declare no conflict of interest.

## Supporting information



Supporting Information

## Data Availability

The data that support the findings of this study are available from the corresponding author upon reasonable request.

## References

[1] M. Prince , A. Wimo , G. M. Guerchet , G.‐C. Ali , Y.‐T. Wu , M. Prina , World Alzheimer. Report 2015: The Global Impact of Dementia: An Analysis of Prevalence, Incidence, Cost and Trends First ed., Alzheimer's Disease International, London, UK 2015.

[2] Y. Huang , L. Mucke , Cell 2012, 148, 1204. 22424230 10.1016/j.cell.2012.02.040 PMC3319071

[3] a) L. Parnetti , L. Gaetani , P. Eusebi , S. Paciotti , O. Hansson , O. El‐Agnaf , B. Mollenhauer , K. Blennow , P. Calabresi , Lancet Neurol. 2019, 18, 573; 30981640 10.1016/S1474-4422(19)30024-9

[4] J. Wang , B. J. Gu , C. L. Masters , Y. J. Wang , Nat. Rev. Neurol. 2017, 13, 612. 28960209 10.1038/nrneurol.2017.111

[5] L. C. Brazaca , I. Sampaio , V. Zucolotto , B. C. Janegitz , Talanta 2010, 210, 120644. 10.1016/j.talanta.2019.120644 31987214

[6] a) R. Somenath , G. Zhiqiang , Nano Today 2009, 4, 318;

[7] a) G. B. Frisoni , M. Boccardi , F. Barkhof , K. Blennow , S. Cappa , K. Chiotis , J. Demonet , V. Garibotto , P. Giannakopoulos , A. Gietl , O. Hansson , K. Herholz , C. R. Jack Jr. , F. Nobili , A. Nordberg , J. Molinuevo , A. U. Monsch , U. Mosimann , A. Padovani , A. Picco , C. Porteri , O. Ratib , L. Saint‐aubert , C. Scerri , P. Scheltens , J. M. Schott , I. Sonni , S. Teipel , P. Vineis , P. J. Visser , et al., Lancet Neurol. 2017, 16, 661; 28721928 10.1016/S1474-4422(17)30159-X

[8] a) S. Lehmann , C. Delaby , G. Boursier , C. Catteau , N. Ginestet , L. Tiers , A. Maceski , S. Navucet , C. Paquet , J. Dumurgier , Front. Aging Neurosci. 2018, 10, 138; 29892221 10.3389/fnagi.2018.00138 PMC5985301

[9] L. M. Shaw , M. Korecka , C. M. Clark , V. M. Y. Lee , J. Q. Trojanowski , Nat. Rev. Drug Discovery 2007, 6, 295. 17347655 10.1038/nrd2176

[10] a) L. O. Tjernberg , J. Näslund , F. Lindqvist , J. Johansson , A. R. Karlström , J. Thyberg , L. Terenius , C. Nordstedt , J. Biol. Chem. 1996, 271, 8545; 8621479 10.1074/jbc.271.15.8545

[11] a) E. K. Samani , M. R. Mofid , M. Malakoutikhah , J. Pep. Sci. 2020, 26, 3227; 10.1002/psc.3227 31845472

[12] R. Tosto , S. Zimbone , G. Sabatino , G. Di Natale , M. L. Giuffrida , M. F. Tomasello , L. Lanzanò , T. Campagna , S. Covaceuszach , G. Vecchio , G. Pappalardo , Chem. Bio. Chem. 2024, 25, 202400431. 10.1002/cbic.202400431 PMC11610669 39382238

[13] a) B. Shui , D. Tao , A. Florea , J. Cheng , Q. Zhao , Y. Gu , W. Li , N. Jaffrezic‐Renault , Biochimie 2018, 147, 13; 29307704 10.1016/j.biochi.2017.12.015

[14] T. A. Enache , A.‐M. Chiorcea‐Paquim , A. M. Oliveira‐Brett , Anal. Chem. 2018, 90, 2285. 29314823 10.1021/acs.analchem.7b04686

[15] M. Ayiania , M. Smith , A. J. R. Hensley , L. Scudiero , J.‐S. McEwen , M. Garcia‐Perez , Carbon 2020, 162, 528.

[16] A. Artemenko , A. Shchukarev , P. Štenclová , T. Wågberg , J. Segervald , X. Jia , A. Kromka , Mat. Sci. Eng. 2021, 1050, 012001.

[17] a) N. S. Clarke , P. G. Hall , Langmuir 1991, 7, 678;

[18] a) F. Zheng , W.‐L. Xu , H.‐D. Jin , X.‐T. Hao , K. P. Ghiggino , RSC. Adv. 2015, 5, 89515;

[19] a) V. Perugini , M. Santin , Sensors 2022, 22, 9561; 36502262 10.3390/s22239561 PMC9736926

[20] P. Supraja , S. Tripathy , R. Singh , V. Singh , G. Chaudhury , S. G. Singh , Biosens. Bioelectr. 2021, 186, 113294. 10.1016/j.bios.2021.113294 33971525

[21] D. Park , J. H. Kim , H. J. Kim , D. Lee , D. S. Lee , D. S. Yoon , K. S. Hwang , Biosens. Bioelectr. 2020, 167, 112505. 10.1016/j.bios.2020.112505 32841782

[22] a) T. Janas , K. Sapon , M. H. B. Stowell , T. Janas , Int. J. Mol. Sci. 2019, 20, 299; 30642129 10.3390/ijms20020299 PMC6359565

[23] a) N. R. Barthélemy , A. Gabelle , C. Hirtz , F. Fenaille , N. Sergeant , S. Schraen‐Maschke , J. Vialaret , L. Buée , C. Junot , F. Becher , S. Lehmann , J Alzheimers Dis. 2016, 51, 1033; 26923020 10.3233/JAD-150962

[24] Y. Xiao , B. Ma , D. McElheny , S. Parthasarathy , F. Long , M. Hoshi , R. Nussinov , Y. Ishii , Nat. Struc Mol. Biol. 2015, 22, 499. 10.1038/nsmb.2991 PMC4476499 25938662

[25] M. L. Giuffrida , F. M. Tomasello , F. Caraci , G. Pandini , G. Pappalardo , F. Attanasio , S. Chiechio , S. Bagnoli , B. Nacmias , S. Sorbi , R. Vigneri , F. N. E. Rizzarelli , A. Copani , Front. Cell. Neurosci. 2015, 9, 297. 26300732 10.3389/fncel.2015.00297 PMC4528168

[26] O. Hansson , K. Blennow , H. Zetterberg , J. Dage , Nat. Aging 2023, 3, 506. 37202517 10.1038/s43587-023-00403-3 PMC10979350

[27] a) I. Batatia , D. P. Kovacs , G. Simm , C. Ortner , G. Csányi , Adv. Neur. Infor. Proc. Syst. 2022, 35, 11423;

[28] O. Crescenzi , S. Tomaselli , R. Guerrini , S. Salvadori , A. M. D'Ursi , P. A. Temussi , D. Picone , Eur. J. Biochem. 2022, 269, 5642. 10.1046/j.1432-1033.2002.03271.x 12423364

[29] A. Santoro , M. Grimaldi , M. Buonocore , I. Stillitano , A. M. D'Ursi , Pharmaceuticals 2021, 14, 732. 34451828 10.3390/ph14080732 PMC8400958

[30] a) S. Xu , C. Zhang , S. Jiang , G. Hu , X. Li , Y. Zou , H. Liu , J. Li , Z. Li , X. Wang , M. Li , J. Wang , Sens. Act. B: Chem. 2019, 284, 125;

[31] G. Di Natale , F. Bellia , M. F. M. Sciacca , T. Campagna , G. Pappalardo , Inorg. Chim Acta 2018, 472, 82.

[32] S. Zimbone , M. L. Giuffrida , G. Sabatino , G. Di Natale , R. Tosto , G. M. L. Consoli , D. Milardi , G. Pappalardo , M. F. M. Sciacca , ACS Chem. Neurosci. 2023, 14, 1126. 36857606 10.1021/acschemneuro.2c00720 PMC10020970

[33] a) S. Baldo , S. Buccheri , A. Ballo , M. Camarda , A. La Magna , M. E. Castagna , A. Romano , D. Iannazzo , F. Di Raimondo , G. Neri , S. Scalese , Sens. Bio‐Sens. Res. 2016, 7, 168;

[34] D. Ricciarelli , S. Filice , G. Calogero , V. Scuderi , S. Boscarino , V. Iacono , F. Ruffino , I. Deretzis , G. Fisicaro , S. Scalese , A. La Magna , ACS Appl. Nano Mat. 2024, 7, 24969.

[35] M. A. Buccheri , D. D'Angelo , S. Scalese , S. F. Spanò , S. Filice , E. Fazio , G. Compagnini , M. Zimbone , M. V. Brundo , R. Pecoraro , A. Alba , Nanotech. 2016, 27, 245704. 10.1088/0957-4484/27/24/245704 27158973

[36] P. A. Vargas‐Rosales , A. D'Addio , Y. Zhang , A. Caflisch , ACS. Phys. Chem Au 2023, 3, 456. 37780539 10.1021/acsphyschemau.3c00021 PMC10540290

