# Supplementary material for: KLVFF Functionalized Graphene Oxide for Aβ42 Peptide Electrical Detection: A Promising Nanomaterial for the Development of Alzheimer's Disease Diagnostic Devices
Source: Small. 2025 Jun 16;21(32):2503488. doi: 10.1002/smll.202503488 (PMC12366246; doi:10.1002/smll.202503488)
Supplement: Supplementary file 1 — Supporting Information [file SMLL-21-2503488-s001.docx]

Supporting Information

KLVFF functionalized graphene oxide for Aβ_42_ peptide electrical detection: a promising nanomaterial for the development of Alzheimer's disease diagnostic devices.

Viviana Scuderi ^1, *^, Rita Turnaturi ^2^, Simona Filice ^1^, Simona Crispi ^1^, Giuseppe Di Natale^2^, Giuseppina Sabatino ^2^, Damiano Ricciarelli ^1^, Giuseppe Fisicaro ^1^, Giuseppe Pappalardo^2^, Antonino La Magna ^1^ and Silvia Scalese ^1^


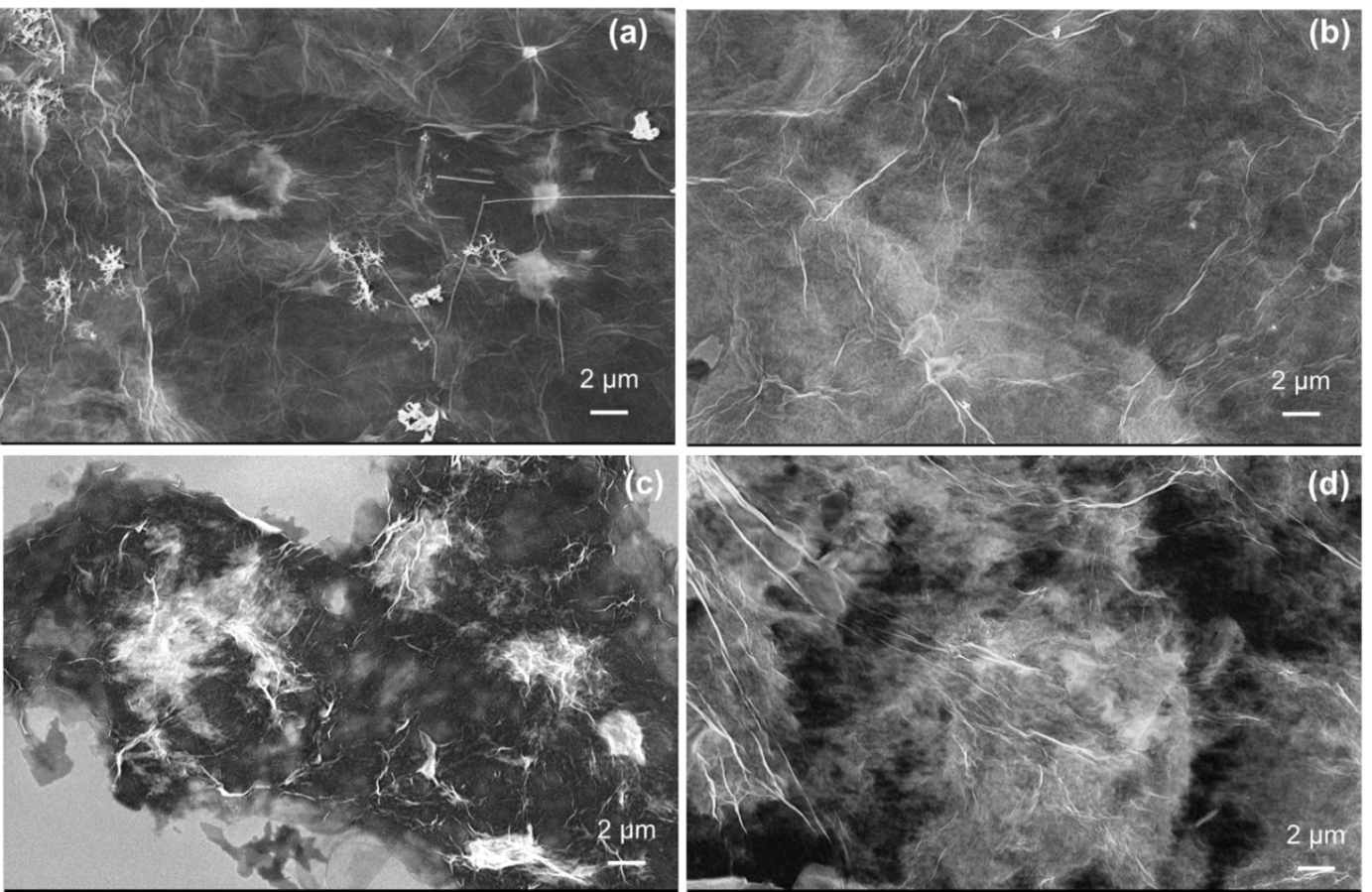


**Figure S1**: SEM images of different GO@peg_4_-KLVFF concentration ratios tested: (a) GO 0.5 mg mL^-1^ + peg_4_-KLVFF 1 µg mL^-1^; (b) GO 0.5 mg mL^-1^ + peg_4_-KLVFF 10 µg mL^-1^; (c) GO 0.5 mg mlL^-1^ + peg_4_-KLVFF 100 µg mL^-1^; (d) GO 0.5 mg mL^-1^ + peg_4_-KLVFF 1000 µg mL^-1^.


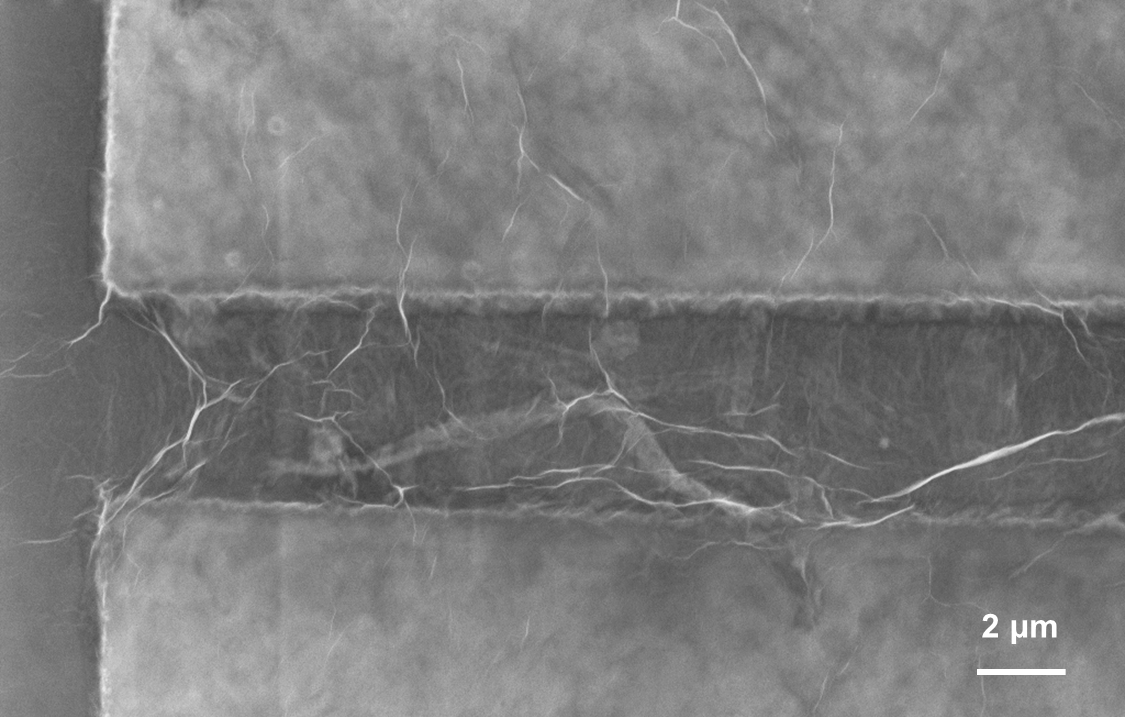


**Figure S2**: SEM images of GO@peg_4_-KLVFF deposited by DEP.


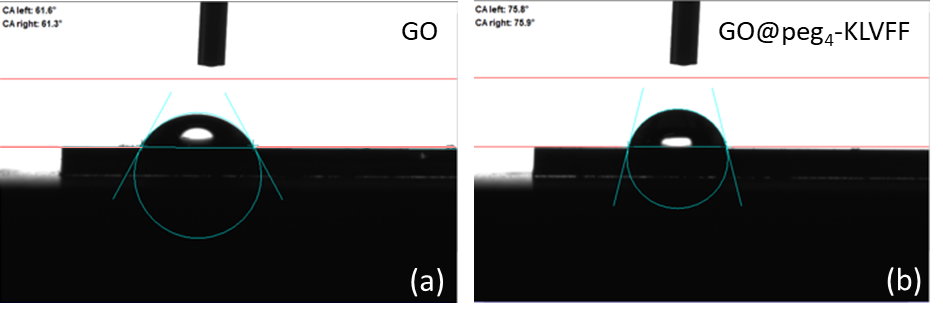


**Figure S3**: Contact angle measurements of (a) GO and (b) GO@peg_4_-KLVFF deposited by DEP.


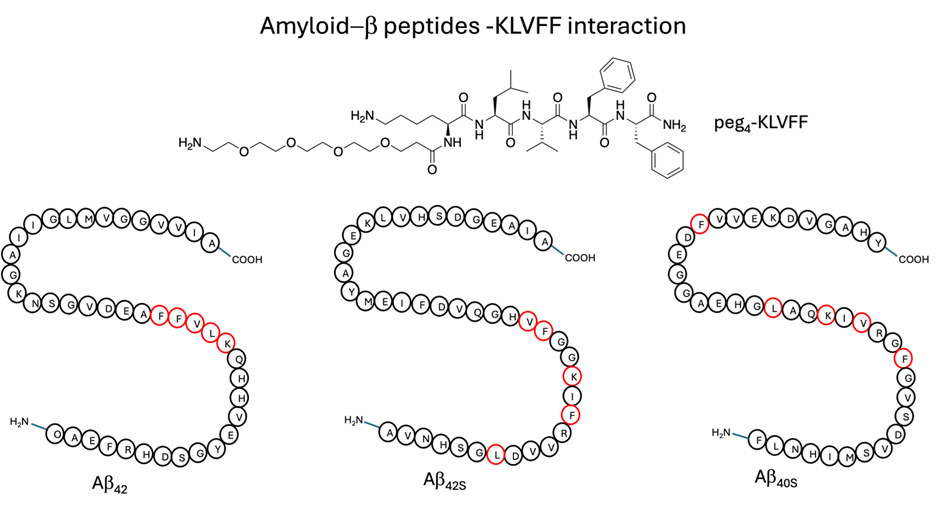


**Figure S4:** Sketch of amyloid-β peptides used in this study (Aβ_1-42_, Aβ_1-42S_, Aβ_1-40S_). Key amino acids involved in the interaction have been highlighted in red.

*Aβ monomerization.* The Aβ peptides were dissolved in TFA (1 mg mL^-1^) and sonicated in a water bath for 10 min. After TFA evaporation under N_2_ stream, 1 mL of hexafluoroisopropanol (HFIP) was added and the resulting peptide solution was incubated at 37 °C for 1h. The peptide solution was dried under a stream of N_2_, and the residual was dissolved in 1 mL of HFIP. To remove the remaining trace of TFA the sample was re-dissolved in 1 mL HFIP, frozen at −80 °C and finally lyophilized overnight.

*Aβ peptides sequences.*

**Amyloid-β (1-42)**

H-Asp-Ala-Glu-Phe-Arg-His-Asp-Ser-Gly-Tyr-Glu-Val-His-His-Gln-Lys-Leu-Val-Phe-Phe-Ala-Glu-Asp-Val-Gly-Ser-Asn-Lys-Gly-Ala-Ile-Ile-Gly-Leu-Met-Val-Gly-Gly-Val-Val-Ile-Ala-OH

DAEFRHDSGYEVHHQKLVFFAEDVGSNKGAIIGLMVGGVVIA

**Scrambled amyloid-β (1-40)**

H-Tyr-His-Ala-Gly-Val-Asp-Lys-Glu-Val-Val-Phe-Asp-Glu-Gly-Gly-Ala-Glu-His-Gly-Leu-Ala-Gln-Lys-Ile-Val-Arg-Gly-Phe-Gly-Val-Ser-Asp-Val-Ser-Met-Ile-His-Asn-Leu-Phe-OH

YHAGVDKEVVFDEGGAEHGLAQKIVRGFGVSDVSMIHNLF

**Scrambled Amyloid-β(1-42)**

H-Ala-Ile-Ala-Glu-Gly-Asp-Ser-His-Val-Leu-Lys-Glu-Gly-Ala-Tyr-Met-Glu-Ile-Phe-Asp-Val-Gln-Gly-His-Val-Phe-Gly-Gly-Lys-Ile-Phe-Arg-Val-Val-Asp-Leu-Gly-Ser-His-Asn-Val-Ala-OHAIAEGDSHVLKEGAYMEIFDVQGHVFGGKIFRVVDLGSHNVA

**Tau 26-44**

H-Gln-Gly-Gly-Tyr-Thr-Met-His-Gln-Asp-Gln-Glu-Gly-Asp-Thr-Asp-Ala-Gly-Leu-Lys-OH

QGGYTMHQDQEGDTDAGLK

*
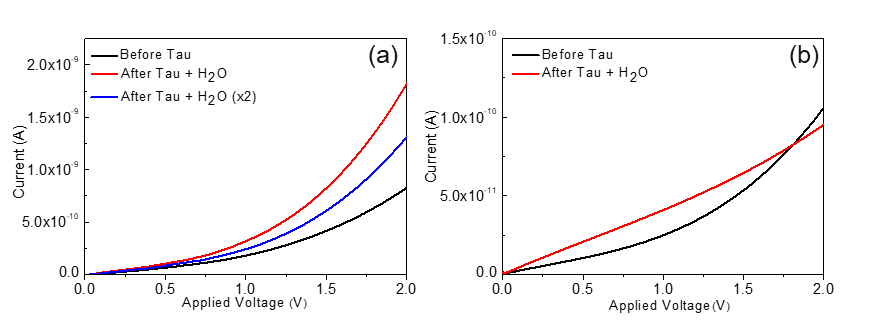
*

**Figure S5**: I-V curves for (a) pristine GO sample before (black line) and after the interaction with Tau 26-44 (9 μM) followed by one (red line), two (blue line) washing processes; (b) GO@peg_4_-KLVFF sample before (black line) and after the interaction with Tau 26-44 (9 μM) and followed by abundant washing in deionized water (red line).


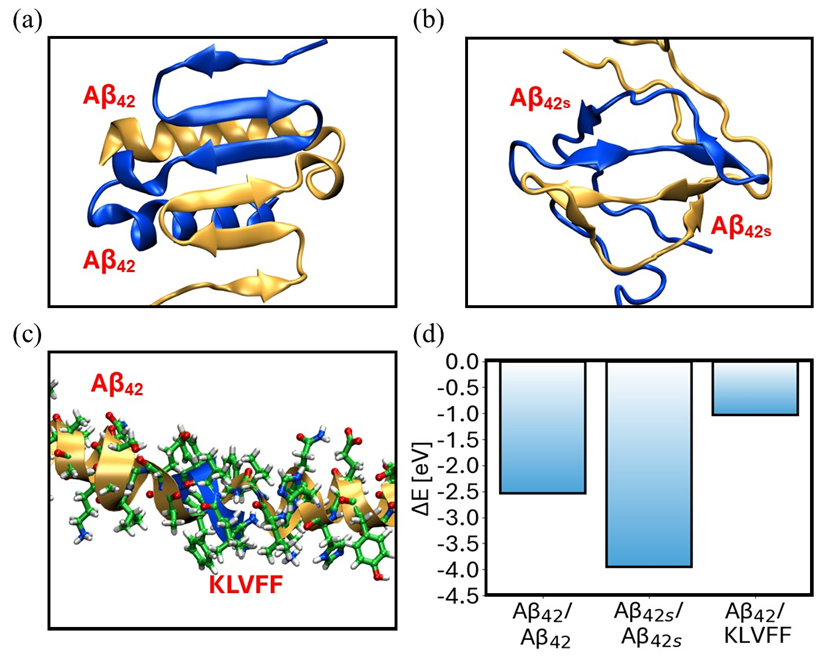


**Figure S6.** Molecular interactions between (a) Aβ_42_ and (b) Aβ_42s_ monomers, and between (c) Aβ_42_ and KLVFF, with corresponding interaction energies in (d).

**Figure S7:** RP-HPLC MS of peg_4_-KLVFF. An UltiMate 3000 Proteomics LC instrument, equipped with a Q-Exactive Orbitrap mass spectrometer (Thermo Fisher Scientific instrument) and controlled by the **Chromeleon** and Xcalibur™ (Thermo Scientific) software), was used for sample analyses. Samples were analysed by gradient elution with A (0.01% formic acid in water) and B (0.01% formic acid in 80% acetonitrile / 20% water) with an EASY-Spray PepMap C18 column (15cm length, 75 μm internal diameter); 100 Å pore size, 3 μm particle size) at a nano-flow rate of 0.3 nLmin^-1^. Samples were injected into the column equilibrated to 95% A and eluted at a flow rate of 0.3 nLmin^-1^: 0 to 5 min isocratic elution at 55% A, linear gradient from 5%–50% B over 17 min, isocratic gradient at 50% B from 17 to 22 min, then isocratic gradient at 50% B from 22 to 27 min. The separation was monitored by ESI-MS. The instrumental parameters for spectra acquired in the positive-ion mode were: spray voltage = 3.5 kV, capillary temperature = 250 °C; m/z range = 200–2000, S-lens RF level = 60 V, Sheath gas = 5, resolving power: 140000 FWHM. The m/z signals were assigned by comparing the simulated and experimental isotopic distribution spectra.

**Table S1.** Variation of the resistance R_0_ (Ohm) as a function of time, of a set of samples stored in a desiccator, in which the GO@peg_4_-KLVFF solution was deposited by DEP. The samples are stable at least for a week and after this time the resistance values start to increase for some of them. Values ​​that are outside the experimental error are highlighted in red.

| Sample | R_0_ (Ohm) | R_0__4days (Ohm) | R_0__7days (Ohm) | R_0__11days (Ohm) |
| --- | --- | --- | --- | --- |
| 119 | (1.5±0.4)E10 | (1.6±0.4)E10 | (1.7±0.4)E10 | (2.0±0.4)E10 |
| 120 | (2.3±0.4)E10 | (2.9±0.4)E10 | (3.3±0.4)E10 | (4.4±0.4)E10 |
| 121 | (1.6±0.4)E10 | (1.6±0.4)E10 | (2.0±0.4)E10 | (2.0±0.4)E10 |

**Table S2.** Variation of the resistance R_0_ (Ohm) as a function of time, of a set of samples stored in a refrigerator at 4°C, in which the GO@peg_4_-KLVFF solution was deposited by DEP. The samples are stable at least for 18 days. Values ​​that are outside the experimental error are highlighted in red.

| Sample | R_0_  (Ohm) | R_0__4days (Ohm) | R_0__7days (Ohm) | R_0__11days (Ohm) | R_0__14days (Ohm) | R_0__18days (Ohm) |
| --- | --- | --- | --- | --- | --- | --- |
| 116 | (2.0±0.4)E10 | (1.2±0.4)E10 | (1.3±0.4)E10 | (1.4±0.4)E10 | (1.6±0.4)E10 | (2.0±0.4)E10 |
| 117 | (1.3±0.4)E10 | (1.1±0.4)E10 | (1.1±0.4)E10 | (1.2±0.4)E10 | (1.3±0.4)E10 | (1.4±0.4)E10 |
| 118 | (1.5±0.4)E10 | (1.2±0.4)E10 | (1.1±0.4)E10 | (1.3±0.4)E10 | (1.4±0.4)E10 | (1.4±0.4)E10 |
